# The Findings of SPECT/CT Concerning Bypass Lymph Circulation in Lymphedema Following Breast Cancer Surgery

**DOI:** 10.3390/healthcare9040471

**Published:** 2021-04-15

**Authors:** Min-Young Lee, Eun-Jung Kong, Dong-Gyu Lee

**Affiliations:** 1 Department of Physical Medicine and Rehabilitation, College of Medicine, Yeungnam University, Daegu 42415, Korea; lmy511@naver.com; 2 Department of Nuclear Medicine, College of Medicine, Yeungnam University, Daegu 42415, Korea; kongej@yu.ac.kr

**Keywords:** lymphedema, breast cancer, axillary lymph, lymphangiography, SPECT

## Abstract

This study aimed to determine whether bypass circulation was present in lymphedema and its effect. This was a retrospective, cross-sectional study. Patients who underwent unilateral breast cancer surgery with axillary lymph node dissection were recruited and underwent single-photon emission tomography/computed tomography (SPECT/CT). SPECT/CT was performed to detect the three-dimensional locations of radio-activated lymph nodes. Patients with radioactivity in anatomical locations other than axillary lymph nodes were classified into a positive group. All patients received complete decongestive therapy (CDT). Exclusion criteria were as follows: History of bilateral breast cancer surgery, cervical lymph node dissection history, and upper extremity amputation. The difference in the upper extremity circumference (cm) was measured at four points: Mid-point of the upper arm, elbow, and 10 and 15 cm below the elbow. Twenty-nine patients were included in this study. Fifteen patients (51.7%) had bypass lymphatic systems on the affected side, six (20.7%) had a bypass lymphatic system with axillary lymph nodes on the unaffected side, and 11 (37.9%) showed new lymphatic drainage. The positive group showed significantly less swelling than the negative group at the mid-arm, elbow, and 15 cm below the elbow. Bypass lymphatic circulation had two patterns: Infraclavicular lymph nodes and supraclavicular and/or cervical lymph nodes. Changes in lymph drainage caused by surgery triggered the activation of the superficial lymphatic drainage system to relieve lymphedema. Superficial lymphatic drainage has a connection through the deltopectoral groove.

## 1. Introduction

Lymphedema is one of the complications that reduces the quality of life of breast cancer survivors [1]. The operative method, axillary lymph node dissection (ALND), radiation therapy, and compliance with compression therapy influence the incidence and severity of lymphedema. Because axillary lymph nodes are the main collectors of lymphatic fluids from the upper extremities, ALND is a critical factor for lymphedema following breast cancer surgery [2,3]. However, lymphangiography following ALND showed activation of the periclavicular lymph node area without the axillary lymph node [4]. These newly formed lymphatics following the operation are known as bypass lymphatic circulation connecting deep and superficial lymphatic drainage [5].

A previous study showed that bypass lymphatic circulation reduced lymphedema in patients who underwent ALND [1]. Even if the main collector of lymph fluid has been removed through ALND, functional bypass lymph circulation has been shown to play a critical role in lymphatic fluid drainage [6]. Previous studies concerning the bypass lymphatic circulation used lymphangiography. However, lymphangiography is a two-dimensional imaging technique; therefore, three-dimensional analysis is not possible. Hence, there is little anatomical information on the bypass circulation.

Single-photon emission tomography/computed tomography (SPECT/CT) is a type of nuclear medicine. SPECT/CT effectively detects the three-dimensional locations of the lymphatic circulation and radio-activated lymph nodes. However, as bypass circulation has not received much attention, only two-dimensional studies, such as lymphangiography, have been used for the differential diagnosis of lymphedema.

Therefore, this study aimed to evaluate the anatomical information of bypass lymph circulation in patients with lymphedema using SPECT/CT. Moreover, the purpose of this study was to determine whether bypass circulation was also present on the unaffected side and its effect on lymphedema.

## 2. Methods

### 2.1. Subjects

This study was conducted retrospectively, and data were collected from an electronic medical record database. The institutional review board approved this study. Breast cancer survivors who met the study inclusion criteria were recruited. All subjects were transferred to the rehabilitation department for subjective and objective symptoms of lymphedema following surgery between March 2019 and January 2020. The inclusion criteria were as follows: (1) Unilateral breast cancer diagnosis and surgery; (2) patients who had axillary lymph node dissection; (3) SPECT/CT having been conducted; and (4) patients who received complete decongestive therapy (CDT). The exclusion criteria were as follows: (1) History of bilateral breast cancer surgery, (2) history of cervical lymph node dissection, and (3) upper extremity amputation.

### 2.2. Circumference Measurements

The circumference of the upper extremities (cm) was measured at four points: The mid-point of the upper arm, elbow, 10 cm below the elbow, and 15 cm below the elbow. The mid-point of the upper arm was measured between the lateral epicondyle of the humerus and the acromion of the shoulder. The elbow level circumference was measured at the lateral and medial epicondyles. The circumferences below the elbow were measured at 10 and 15 cm below the lateral epicondyle of the humerus. The difference in the circumference (cm) was calculated as the difference between the unaffected and affected sides of the upper extremities.

### 2.3. SPECT/CT

The patients received four doses of 37 MBq of Tc-99m phytate (unfiltered) in 0.1-mL volumes injected intradermally into the first and second interdigital web spaces of the dorsum of each hand. The patients were immediately encouraged to perform repeated gripping exercises for 5 min to induce lymphatic flow in the upper limbs. Using a dual-head SPECT/CT scanner (Optima NM/CT 640; General Electric Company, Boston, MA, United States), early (5 min) planar scintigraphy and late (120 min) planar scintigraphy with SPECT/CT images were performed. The patient’s arms were down by their sides, and planar images were acquired at a speed of 14 cm/min using a 256 × 1024 matrix from the wrist to the neck. SPECT was performed in the axilla and upper arm area using a 128 × 128 matrix in the step and shoot mode (20 s/step, 3° angle). The scanner was equipped with a large field of view and a low-energy high-resolution collimator. SPECT images were reconstructed using an iterative ordered subset expectation maximization (OSEM) algorithm with two iterations and 10 subsets with a slice thickness of 4.42 mm. CT for image co-registration and attenuation correction was performed using a 512 × 512 matrix, 30 mAs, a voltage of 120 kVp, and a 2.5-mm slice thickness at the same position without contrast agent enhancement. According to the bypass lymph circulation on the two-hour delay shot, SPET/CT findings were classified into two groups: A positive and a negative group. If radioactivity was seen in anatomical locations other than the axillary lymph nodes on the affected side, it was defined as the presence of bypass lymph circulation. One nuclear medicine physician, who was blinded to the clinical information, interpreted the uptake patterns of the SPET/CT images. The positive and negative groups were assigned according to the presence or absence of bypass lymph circulation on the affected side on SPECT/CT images.

### 2.4. Statistical Analysis

Data input and statistical analyses were performed using SPSS ver. 25.0 (SPSS Inc., Chicago, IL, USA). According to the bypass lymphatic circulation detected by SPECT/CT, the severity of lymphedema was also analyzed using the Mann-Whitney U-test. Statistical significance was set at *p* < 0.05.

## 3. Results

**Figure 1:**
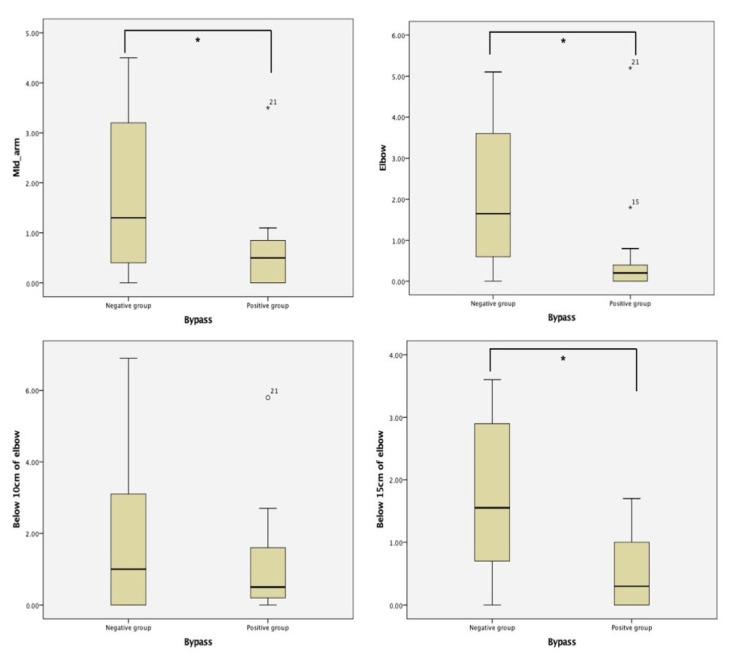
The difference in circumference on the upper extremities at four points according to bypass circulation positive and negative groups. The four points of the upper extremity are the mid-point of the upper arm, elbow, 10 cm below the elbow, and 15 cm below the elbow, respectively. The negative group shows significant severe lymphedema compared to the positive group at the mid-arm, elbow, and 15 cm below the elbow. * *p* < 0.05.

Twenty-nine patients were included in this study (Table 1). Fifteen patients (51.7%) showed bypass lymphatic systems on the affected side. In the negative group, the mean circumference at the mid-arm, elbow, 10 cm below the elbow, and 15 cm below the elbow was 1.8 ± 1.58, 1.97 ± 1.73, 1.82 ± 2.19, and 1.71 ± 1.24, respectively (Figure 1). In the positive group, the mean circumference at the mid-arm, elbow, 10 cm below the elbow, and 15 cm below the elbow was 0.6 ± 0.83, 0.62 ± 1.27, 1.2 ± 1.45, and 0.44 ± 1.57, respectively. The positive group showed statistically significantly less swelling than the negative group at the mid-arm, elbow, and 15 cm below the elbow.

Six patients (20.7%) showed a bypass lymphatic system with axillary lymph nodes on the unaffected side. These patients had axillary lymph nodes and bypass circulation together for lymphatic drainage from the upper extremities (Table 2). Among them, four patients still showed bypass lymphatic circulation on the affected side following ALND. However, two patients did not show bypass lymphatic circulation on the affected side following surgery. Twenty-three patients did not have bypass lymphatic circulation on the unaffected side. Eleven (37.9%) patients had new lymphatic drainage connections by bypassing the axillary lymph nodes as deep lymphatic collectors.

**Figure 2:**
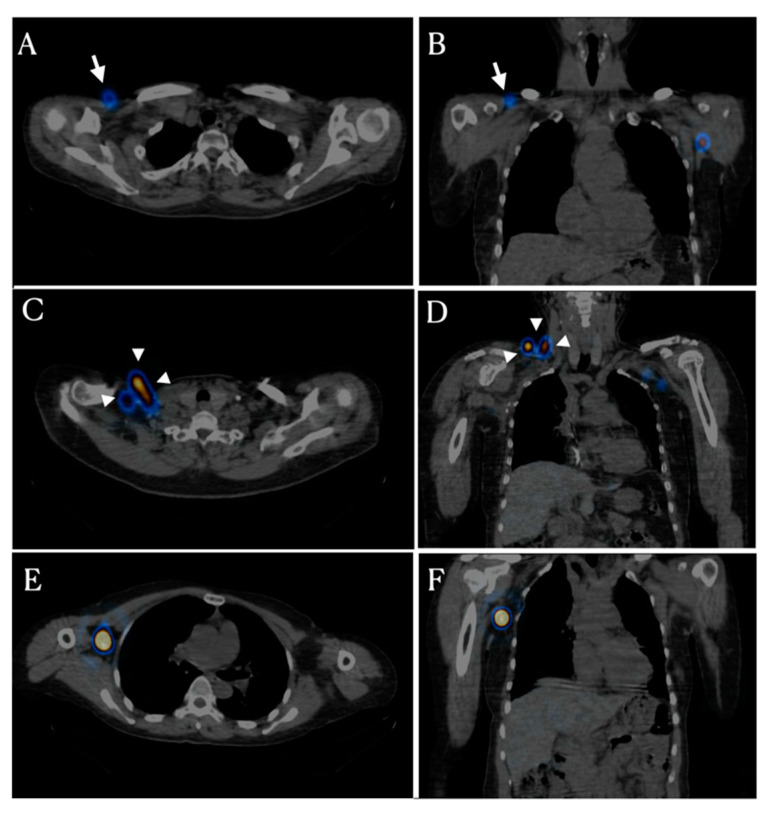
SPECT/CT findings of two types of bypass circulation. (**A**,**B**) The infraclavicular type shows radioactivity on the deltopectoral groove (arrow) below the right clavicle. (**C**,**D**) The supraclavicular type (arrowhead) shows radioactivity around the right clavicular fossa above the clavicle. (**E**,**F**) The right axillary lymph nodes are activated within the axillary fossa. However, there are no radioactivated axillary lymph nodes on left side following axillary lymph node dissection.

Bypass lymphatic circulation has two patterns. The first pattern is infraclavicular lymph node activation (Figure 2A,B). Thirteen patients had infraclavicular lymph node activation in the affected axillary area. These lymph nodes are located from the deltopectoral groove to the infraclavicular area, which is known as the route of the superficial lymphatic pathway in the upper arm. The second pattern is activation of the supraclavicular and/or cervical lymph node that is located in the fascia within the supraclavicular fossa just lateral to the clavicular head (Figure 2C,D). Two patients showed supraclavicular lymph node activation.

## 4. Discussion

Patients with bypass circulation showed less lymphedema than patients without bypass circulation. Among the 23 patients without bypass circulation on the unaffected side, 11 patients (37.9%) had a new bypass lymphatic circulation on the affected side following ALND. Therefore, the removed axillary lymph nodes promoted the patent and/or new lymphatic connections between the deep and superficial lymph correctors. This bypass lymphatic circulation improved the lymphatic drainage from the upper extremities. Six patients (20.7%) had bypass circulation on the unaffected side. However, two patients did not have bypass circulation on the affected side following ALND.

Axillary lymph nodes are the primary sites of lymphatic drainage of the upper extremities. Therefore, ALND has been a critical factor for lymphedema following breast cancer surgery [7]. Although the need for ALND has decreased, ALND has been used to reduce the local recurrence of breast cancer in axillary lymph nodes [8,9]. Moreover, bypass circulation reduces lymphedema following ALND [4]. Therefore, it is meaningful to study bypass circulation as a replacement for axillary lymph nodes.

We wanted to know whether bypass circulation plays a role in the drainage of hand and forearm lymph fluid. SPECT/CT findings can show the main lymph node receiving afferent lymphatics from the forearm and hand. SPECT/CT was used to confirm the anatomical pathways of bypass circulation. The radioactivity of the unaffected side was estimated to confirm that the bypass circulation was already functioning. Twenty-three patients (79.3%) had radioactivity only in the deep collectors and axillary lymph nodes. Six patients (20.7%) had an activated superficial lymph node with axillary lymph nodes on the unaffected side. Patients without bypass lymphatic circulation on the unaffected side showed new bypass lymph node activation around the shoulder girdle.

There are two types of bypass lymphatic circulation. The first is infraclavicular lymph node activation. Lymph nodes of the infraclavicular area through the deltopectoral groove are superficial collectors that drain lymphatic fluid from the deltoid and lateral upper arm [10]. Deltopectoral lymph nodes are superficially located on the deltopectoral groove between the deltoid and pectoral muscles [11]. The activity of the superficial collector, infraclavicular lymph nodes including deltopectoral lymph nodes following ALND is a response to the absence of the deep collector, the axillary lymph node. Therefore, the bypass circulation through the superficial collector explains that the lymphatic system is a dynamic structure through lymphangiogenesis or activation of latent connections [12,13].

The second-pass circulation was through the supraclavicular lymph nodes. The supraclavicular lymph node receives lymph from the superficial and deep collectors of the upper arm. Based on the removed state of the axillary lymph node, it is speculated that supraclavicular lymph nodes receive lymph from the superficial lymphatic circulation through the deltopectoral and subclavicular lymph nodes. Some subjects do not have a deltopectoral lymphatic drainage pathway from the upper arm [10]. Moreover, bypass circulation of the deltopectoral pathway passes to the apical axillary nodes and/or the supraclavicular lymph nodes [14]. Therefore, if the deltopectoral lymph node is inactive or absent, the supraclavicular lymph nodes can act as the main collector of lymph in the bypass circulation through the superficial lymph collector.

Two patients lost bypass circulation following ALND, as bypass circulation was activated on the unaffected side. The dorsolateral upper arm bundle (DUAB) has a connection variant between the medial upper arm bundle [15]. Long-type connections of DUAB receive lymph from the dorsolateral upper arm and forearm territory. Therefore, if deep axillary collectors are removed, the DUAB can act as a bypass circulation route. However, the short type has a connection around the axillary area between the DUAB and medial bundle of the forearm. Moreover, occasionally the short type discharges directly into the medial upper arm bundle and the deep axillary collector. Level III ALND is located between the costoclavicular ligament of Halsted and the medial border of the pectoralis minor, which is close to the apical and subclavicular lymph nodes [16,17]. Therefore, we speculated that the connection between superficial bypass circulation and periclavicular lymph nodes was removed through ALND.

One case report emphasized the importance of bypass circulation following ALND. This case reported that the chemotherapy port in the deltopectoral groove provoked lymphedema [18]. The deltopectoral groove is the pathway of bypass circulation. Chemotherapy port insertion can cause dysfunction of bypass circulation while maintaining lymphatic drainage. Therefore, preoperative PET/CT can evaluate the pathway and the condition of the lymphatic circulation in patients who have undergone ALND.

This study had limitations. First, every included patient presented lymphedema symptoms and was referred to the rehabilitation department for treatment. Therefore, there were no data on patients without symptoms. If breast cancer survivors following ALND did not present with lymphedema symptoms, they could have a functional bypass circulation on the affected side. This study aimed to investigate the new formation of bypass circulation. Despite this limitation, this study provided actual evidence of dynamic changes in the lymphatic system following ALND. Second, we did not correct the variance in the effect of complete decongestive therapy. Despite adequate education and guidance on the treatment, the compliance with and skill of applying the compression bandage differs according to the patients and caregivers. Therefore, quantitative analysis of the effects of complete decongestive therapy is challenging. However, all included patients underwent the same course of rehabilitation teaching courses.

Previously, it was reported that superficial and deep lymph collectors were independent in normal anatomy [19]. However, in this study, after clearance of axillary lymph nodes, the normal anatomy was changed to maintain lymph drainage. In conclusion, dynamic changes in the lymphatic system following ALND can activate the connection between the deep and superficial lymphatic drainage bundles. These changes influence effective treatment outcomes post lymphedema. Further research is needed to determine which factors, including complete decongestive therapy, can affect reconnection or lymphangiogenesis of the lymphatic pathway.

## Figures and Tables

**Table 1:** Demographic data.

		Total	Negative Group	Positive Group	*p*-Value
Age		55.34 ± 10.38	56.71 ± 10.04	54.06 ± 10.88	0.72
Duration (months)		33.75 ± 42.41	56.60 ± 26.86	26.86 ± 23.02	0.79
Side (Right:Left)		13:16	6:8	7:8	0.83
Mean circumference difference (cm)	Mid-arm	1.20 ±1.38	1.8 ± 1.58	0.6 ± 0.83	0.03 *
	Elbow	1.28 ± 1.67	1.97 ± 1.73	0.62 ± 1.27	0.01 *
	Below 10 cm of elbow	1.47 ± 1.88	1.82 ± 2.19	1.2 ± 1.45	0.65
	Below 15 cm of elbow	1.09 ± 1.12	1.71 ± 1.24	0.44 ± 1.57	0.00 *

Values are presented as the mean ± standard deviation, * *p* < 0.05.

**Table 2:** Occurrence of bypass lymphatic nodes on the affected and unaffected side.

		Bypass Circulation on Affected Side	
		Negative Group (14)	Positive Group (15)
Bypass circulation on unaffected side	Negative group (23)	12 (41.4%)	11 (37.9%)
	Positive group (6)	2 (6.9%)	4 (13.8%)

## Data Availability

The datasets used and/or analyzed during the current study are available from the corresponding author upon reasonable request.

## References

[1] Kim J.B., Lee D.G. (2020). Findings of lymphoscintigraphy and the severity of lymphedema according to the extent of axillary lymph node dissection. Asian J. Surg..

[2] Abass M.O., Gismalla M.D., Alsheikh A.A., Elhassan M.M. (2018). Axillary lymph node dissection for breast cancer: Efficacy and complication in developing countries. J. Glob. Oncol..

[3] Roses D.F., Brooks A.D., Harris M.N., Shapiro R.L., Mitnick J. (1999). Complications of level I and II axillary dissection in the treatment of carcinoma of the breast. Ann. Surg..

[4] Kang S.H., Lee D.G. (2020). Periclavicular Lymph Node Activation Maintains the Lymphatic Circulation of Upper Extremity Following Breast Cancer Surgery with Axillary Lymph Node Dissection. Lymphat. Res. Biol..

[5] Suami H., Koelmeyer L., Mackie H., Boyages J. (2018). Patterns of lymphatic drainage after axillary node dissection impact arm lymphoedema severity: A review of animal and clinical imaging studies. Surg. Oncol..

[6] Szuba A., Chachaj A., Koba-Wszedybylb M., Hawro R., Jasinski R., Tarkowski R., Szewczyk K., Bebenek M., Forgacz J., Jodkowska A. (2011). Axillary lymph nodes and arm lymphatic drainage pathways are spared during routine complete axillary clearance in majority of women undergoing breast cancer surgery. Lymphology.

[7] Sakorafas G.H., Peros G., Cataliotti L., Vlastos G. (2006). Lymphedema following axillary lymph node dissection for breast cancer. Surg. Oncol..

[8] Veronesi U., Paganelli G., Galimberti V., Viale G., Zurrida S., Bedoni M., Costa A., De Cicco C., Geraghty J.G., Luini A. (1997). Sentinel-node biopsy to avoid axillary dissection in breast cancer with clinically negative lymph-nodes. Lancet.

[9] Sávolt Á., Péley G., Polgár C., Udvarhelyi N., Rubovszky G., Kovacs E., Győrffy B., Kasler M., Matrai Z. (2017). Eight-year follow up result of the OTOASOR trial: The optimal treatment of the axilla–surgery or radiotherapy after positive sentinel lymph node biopsy in early-stage breast cancer: A randomized, single centre, phase III, non-inferiority trial. Eur. J. Surg. Oncol..

[10] Johnson A.R., Granoff M.D., Suami H., Lee B.T., Singhal D. (2020). Real-Time Visualization of the Mascagni-Sappey Pathway Utilizing ICG Lymphography. Cancers.

[11] Suami H., Scaglioni M.F. (2018). Anatomy of the Lymphatic System and the Lymphosome Concept with Reference to Lymphedema. Semin. Plast. Surg..

[12] Suami H. (2020). Anatomical Theories of the Pathophysiology of Cancer-Related Lymphoedema. Cancers.

[13] Suami H., Scaglioni M.F., Dixon K.A., Tailor R.C. (2016). Interaction between vascularized lymph node transfer and recipient lymphatics after lymph node dissection—A pilot study in a canine model. J. Surg. Res..

[14] Susan S. (2015). Gray’s Anatomy: The Anatomical Basis of Clinical Practice.

[15] Földi M., Földi E., Strößenreuther R., Kubik S. (2012). Földi’s Textbook of Lymphology: For Physicians and Lymphedema Therapists.

[16] Joshi S., Noronha J., Hawaldar R., Kundgulwar G., Vanmali V., Parmar V., Nair N., Shet T., Badwe R. (2019). Merits of Level III Axillary Dissection in Node-Positive Breast Cancer: A Prospective, Single-Institution Study From India. J. Glob. Oncol..

[17] Ecanow J.S., Abe H., Newstead G.M., Ecanow D.B., Jeske J.M. (2013). Axillary staging of breast cancer: What the radiologist should know. Radiographics.

[18] Turfe Z., Pettinga J., Leduc O., Leduc A., Komorowska-Timek E. (2016). Chemotherapy port related lymphedema after axillary lymph node dissection. Breast.

[19] Suami H., Kato S. (2018). Anatomy of the lymphatic system and its structural disorders in lymphoedema. Lymphedema.

